# Extraordinary Case of Varix

**Published:** 1844-02-10

**Authors:** 


### EXTRAORDINARY CASE OF VARIX.

A woman, pregnant for the fifth time, presented very large varices on her legs at the end of the fourth week. Soon afterwards they extended up the abdomen, and two months before her confinement they had even spread up to her arms, breasts, neck, and scalp, so that there were more than fifty large knots on the upper part of the body. All motion was painful, on account of the friction and pressure attending it.

A regulated diet, repeated purgatives, small bleedings, the applications of compression, and the horizontal position, were the remedies prescribed, which gave some relief, although but little.

The child was born without much difficnlty, but died shortly afterwards. The mother rapidly sunk, though the hæmorrhage was not considerable. The uterus contracted little, and the accoucheur discovered, on examination, a mole, which he did not like to remove, on account of the weakness of the patient. She died shortly afterwards, but a post-mortem examination was refused.—*Provin. Med. Journal, from Med. Zeitung.*

